# Atypical Antipsychotic Lumateperone Effects on the Adrenal Gland With Possible Beneficial Effect of Quercetin Co-administration

**DOI:** 10.3389/fphys.2021.674550

**Published:** 2021-06-29

**Authors:** Hala El-Haroun, Suzy Fayez Ewida, Wael M. Y. Mohamed, Manar Ali Bashandy

**Affiliations:** ^1^Department of Histology, Faculty of Medicine, Menoufia University, Shebin El-kom, Egypt; ^2^Department of Medical Physiology, Faculty of Medicine, Menoufia University, Shebin El-kom, Egypt; ^3^Department of Clinical Pharmacology, Faculty of Medicine, Menoufia University, Shebin El-kom, Egypt; ^4^Department of Basic Medical Science, Kulliyyah of Medicine, International Islamic University Malaysia (IIUM), Pahang, Malaysia; ^5^Department of Anatomy Faculty of Medicine, Menoufia University, Shebin El-kom, Egypt

**Keywords:** atypical antipsychotics, lumateperone, hypothalamic pituitary adrenal axis, adrenal gland, depressive like-behavior, quercetin

## Abstract

Schizophrenia remains one of the most chronic and highly disabling mental disorders. Lumateperone is a recent FDA-approved atypical antipsychotic drug for the treatment of schizophrenia. However, the internal FDA pathologist raised concerns regarding pigment deposition associated with degeneration in different tissue in animal studies with lumateperone treatment. The adrenal gland may be implicated in lumateperone side effects, and quercetin may have the ability to fulfill this treatment gap. To prove this hypothesis, 40 male guinea pigs were used and divided into four groups; control, quercetin-treated, lumateperone-treated, and quercetin/lumateperone cotreated orally for 28 consecutive days. Behavioral forced swim (FST) and open field (OF) tests were done at the end of treatment. Retro-orbital blood samples were taken to assess hormones: adrenocorticotropic hormone (ACTH), cortisol, dehydroepiandrosterone acetate (DHEA), and aldosterone, along with an assessment of oxidative stress parameters: malondialdehyde (MDA), glutathione (GSH), and superoxide dismutase (SOD). Adrenal glands were extracted for histopathological assessment with H&E, Mallory trichome staining, immunostaining, and electron microscopy studies. Lumateperone-treated group showed a significant reduction in the activity in FST and OF with histopathological deterioration in adrenal secretory function and structure and increased expression of interleukin-6 (IL-6), CASPASE-3, collagen deposition, and decreased proliferating cell nuclear antigen (PCNA). Cytoplasmic vacuolation, pyknosis of the nuclei, increase in the lysosome, lipofuscin pigment, and cellular infiltration with diminishing in the number of secretory granules could all be observed in lumateperone-treated group. Coadministration of quercetin and lumateperone showed improvement of the previously deteriorated parameters. Quercetin had a prophylactic effect against lumateperone depressive-like effect on animal behavior and its possible adrenal damage.

**Graphical Abstract:**
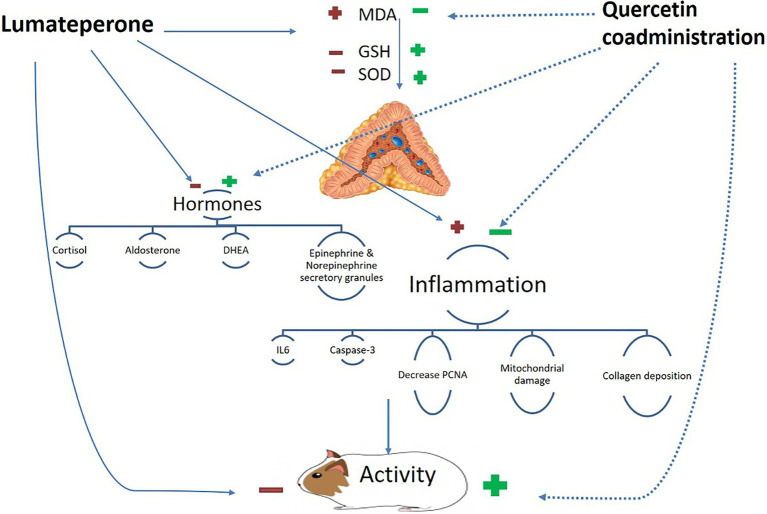
Conceptual framework for the proposed mechanism of action of coadministration of quercetin and lumateperone.

Conceptual framework for the proposed mechanism of action of coadministration of quercetin and lumateperone.

## Introduction

Schizophrenia (SCZ) is a serious psychiatric condition associated with hallucinations or delusions coupled with disorganized speech, disorganized thinking, or negative symptoms present for at least 6 months (Andreasen and Olsen, 1982; Orrico-Sánchez et al., 2020). Negative symptoms include lack of expression, significant reduction in speech/communication, lack of motivation, and decreased social drive (American Psychiatric Association, 1994; Leo and Regno, 2000). About 20 million people worldwide were affected with SCZ (GBD, 2017), with higher susceptibility for early death two to three times more than the general population (Laursen et al., 2014). It is a chronic disorder that requires a lifetime treatment, the mainstay of which is antipsychotics (Cooper and Gupta, 2020). First-generation antipsychotics are nonspecific in their action with dystonia, parkinsonism, and the occurrence of Tardive Dyskinesia as serious side effects. On the other hand, second-generation antipsychotics target more specific dopamine and often serotonin receptors with fewer dystonic side effects; however, they come with additional concern with the development of metabolic syndrome (Meltzer and McGurk, 1999; Harvey et al., 2016; Remington et al., 2016).

Lumateperone is a mechanistically FDA-approved novel agent for the treatment of SCZ (Blair, 2020). It is also being tested in clinical trials to treat the bipolar depressive disorder and behavioral agitation associated with Alzheimer’s disease (Correll et al., 2020; Kantrowitz, 2020). Lumateperone can effectively treat positive symptoms, negative symptoms, and cognitive dysfunction in SCZ (Edinoff et al., 2020). There are several postulated mechanisms of action for lumateperone, including serotonin 5-HT2A receptor antagonism, a partial agonist of presynaptic dopamine D2 receptor and postsynaptic antagonist (Snyder et al., 2015), a modulator of glutamate at the level of D1 receptor as well as serotonin reuptake inhibitor (Correll et al., 2020). It has off-target antagonism at alpha-1 receptors without significant antihistaminic or antimuscarinic properties [Caplyta (lumateperone), 2018]. Compared with other antipsychotic agents, lumateperone possesses a few unfavorable mild side effects such as sedation, fatigue, somnolence, constipation, and dry mouth (Correll et al., 2020). However, the internal FDA pathologist raised concerns regarding pigment deposition associated with lumateperone deposition in different tissues in experimental animals [Caplyta (lumateperone), 2018].

It is noteworthy to mention that the adrenal gland has a big role in the prognosis of SCZ (Sheard, 1958). In a patient with psychosis, several studies reported hyperactivation of the hypothalamic–pituitary–adrenal (HPA) axis and immune system (Handley et al., 2016) that may lead to brain structure and function abnormalities found in psychosis (Zajkowska and Mondelli, 2014). Additionally, atypical antipsychotics impact cortisol release and may induce adrenal impairment (Gupta and Mohanty, 2020).

Quercetin is a plant-origin polyphenolic flavonoid compound (Rauf et al., 2018). It is present in various fruits and vegetables such as red grapes, apples, broccoli, onions, dill, cilantro, capers, lovage, berries, and kales (Almeida et al., 2018). It shows various pharmacological effects as antioxidant, anti-viral, anti-cancer, anti-apoptotic, and anti-inflammatory (Amidi et al., 2019). The antioxidant property of quercetin represents the cornerstone for prevention and treatment of several diseases like cardiovascular disease, diabetes, cancer, neurodegenerative disorders, asthma, allergies, gastritis, chronic inflammation, obesity, osteoporosis, viral and bacterial diseases (David et al., 2016; Liao et al., 2018; Saleh et al., 2018; Fanunza et al., 2020).

Hence, our study was designed to investigate the possible impact of lumateperone alone or with quercetin on behavior and adrenal gland structure and functions using behavioral, biochemical, and immuno-histochemical assessment techniques in guinea pigs.

## Materials and Methods

### Experimental Animals

Forty adult male guinea pigs were chosen in this experiment aged 2–3 months. Their average weight ranged from 400 to 450 g. We used only male animals to avoid the confounder effect of female sex hormones on the biochemical analysis. These animals were purchased from the Medical Research Institute, animal house, Alexandria University, Egypt. Before starting the study, the guinea pigs were acclimatized to laboratory conditions and room temperature (28–32°C) for 10 days. They were housed in metal cages (five animals/cage) and retained under appropriate laboratory conditions (12 h/light and 12 h/dark). The animals had free access to a standard pellet diet, water, and *ad libitum*. Animal care and experimental protocols were approved by Institutional Animal Care and Use Committee (IACUC), Menoufia University, Egypt (MUFS/F/HI/2/20).

### Drugs and Chemicals

Lumateperone tosylate was obtained from Sigma-Aldrich (St. Louis, MO) as a fine pure powder. The dose for an adult human is 42 mg/day for 28 days (Correll et al., 2020). Subsequently, we converted human dose to animal dose according to Shin et al. (2010); thus, the estimated used dose was 3.2 mg/kg/day. Thirty-two milligram was dissolved in dimethyl sulfoxide (DMSO; 1 ml) and diluted in saline to obtain a 10 ml clear solution for oral administration using oral gavage. Quercetin 500-mg capsule dietary supplements (std. 95% quercetin from *Sophora japonica* leaf extract) were obtained from Toniiq LLC (Chicago, IL). The estimated dose used was 50 mg/kg/day (Zhang et al., 2020). The capsule was dissolved in DMSO (1 ml) and diluted in saline to obtain a 10-ml clear solution for oral administration *via* oral gavage.

### Experimental Design

**Figure 1:**
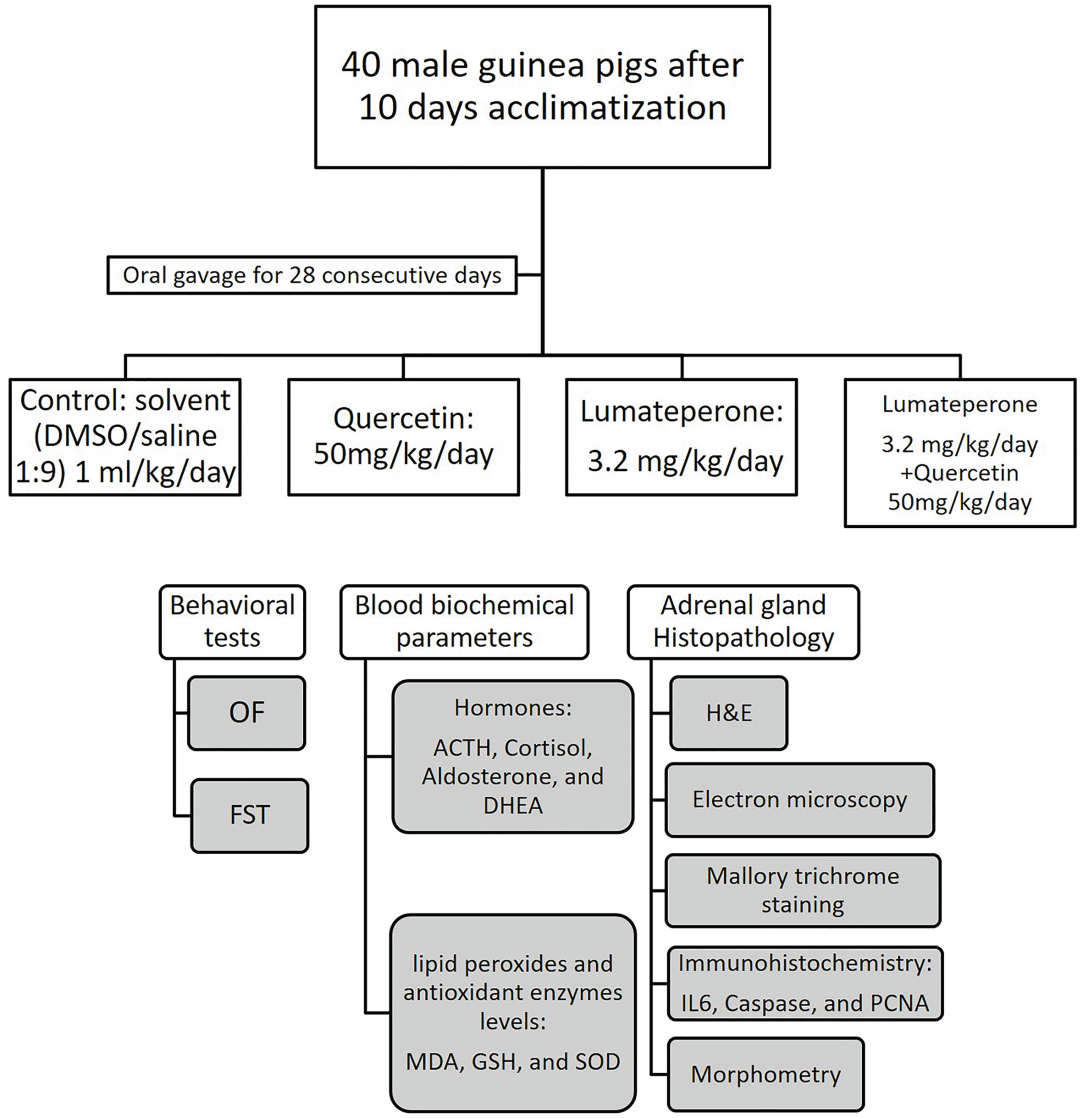
Study design diagram illustrating the groups of animals and the behavioral, biochemical, and histopathological tests done. Open field (OF), forced swim test (FST), hematoxylin and eosin (H&E).

A total of 40 adult male guinea pigs were used in this study, considering 20% dropouts. Reasons for considering 20% dropouts are due to possible performance outliers and potential deaths. The sample size of this study calculated using G*Power software Version 3.1.9.4. (Germany) with a 90% power, 5% significance level, and an effect size of 1.5, the sample sizes required were 10 animals per group. As shown in Figure 1 animals were categorized into four equal groups with 10 animals per group: Group I act as the control group. Animals in this group received 1 ml/kg/day of the solvent mixture (DMSO/saline 1:9 ratio) *via* oral gavage for 28 consecutive days. Group II, in which animals received quercetin with 50 mg/kg/day dissolved in solvent (Zhang et al., 2020) by oral gavage for 28 consecutive days. Group III with animals treated with lumateperone 3.2 mg/kg/day dissolved in the solvent, administered by oral gavage for 28 consecutive days (Shin et al., 2010; Correll et al., 2020). Finally, Group IV, in which animals received 50 mg/kg/day of quercetin and 3.2 mg/kg lumateperone by oral gavage for 28 consecutive days. Animals were weighted routinely every day throughout the experiment, with no significant weight loss detected.

**Figure 2:**
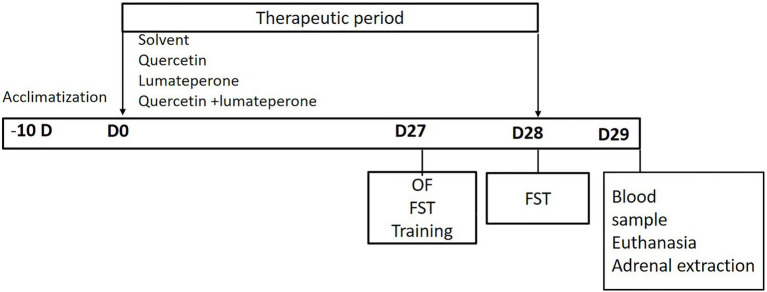
Study timeline diagram illustrating the duration of acclimatization of animals 10 days before the start of therapeutic intervention (−10D), the duration of therapeutic intervention from day 0 (D0) to day 28 (D28), and the timing of different study tests; open field test (OF) on day 27 (D27), Forced swim test (FST) training session on D27, FST on day 28 (D28), blood sample and animal euthanasia for adrenal extraction on day 29 (D29).

On day 27 of the experiment, animals underwent an open field test. This was followed by a forced swim test a day later. Blood samples (3 ml) were then collected from the retro-orbital region for biochemical assessment of adrenocorticotropic hormone (ACTH), cortisol, dehydroepiandrosterone acetate (DHEA), aldosterone, malondialdehyde (MDA), superoxide dismutase (SOD), and glutathione (GSH). Then, the animals were anesthetized using ether and sacrificed by quick cervical decapitation. The adrenal gland, the small yellow gland embedded in fat which is located adjacent to the superior pole of each kidney, was dissected and removed from right and left sides and then was subjected to different histopathological studies (Figures 1, 2).

### Behavioral Tests

#### Forced Swim Test

Forced swim test was done as described previously by Wicke et al. (2007). Shortly, guinea pigs were transferred individually in a metallic cage for a five-minute swimming training session a day before the actual forced swim test. The swim test was done using a locally fabricated plexiglass cylinder (Inner diameter 20 cm, height 40 cm) filled with 21 cm of water maintained at 30 ± 1°C temperature in a quiet, isolated room. On the next day, the animal swimming behavior was assessed manually for 5 min. The total activity per 5-min session and immobility time were recorded manually by a blinded observer (Carlezon et al., 2002). The water in the cylinder was replaced for each animal.

#### Open Field Test

Open field test was used to assess the locomotor activity and the exploratory behavior in Male guinea pigs, as explained before by Braszko et al. (1987). The locally designed open field apparatus consisted of a plexiglass square tank (100 cm × 100 cm × 50 cm). The floor of the area was divided equally into 25 squares. Each guinea pig was placed in the center and left to explore the tank for 5 min. A blinded observer with a video camera was recording the animal behavior. The horizontal locomotion (number of crossed squares) was counted manually. After each test, the arena was cleaned with 90% alcohol solution to remove any odor from the previous animal to avoid smell cues.

### Biochemical Studies

By ending the experiment after 28 days, blood samples were collected from the plexus of veins in the retro-orbital region using a capillary pipette. The collected samples were retained in ice-cooled centrifugal tubes and centrifuged (1,700×*g*, 10 min, 4°C). The serum collected was stored at −80°C. The following biochemical measurements were done for each blood sample:

#### Serum Cortisol, ACTH, Aldosterone, and DHEA Hormones Levels

Kits were purchased from antibodies-online GmbH Company, Egypt, to measure serum cortisol level (Herrero et al., 2007) and serum ACTH (Makrigiannakis et al., 2007). Aldosterone (ng/dL) Immunotag™ Aldosterone EISA Kit, Bioscience, Geno Technology Inc. (St. Louis, MO) and estimated according to Lv et al. (2015). DHEA (dehydroepiandrosterone) level (ng/dL) in serum was determined by ELISA assay kits (enzyme-linked immunoassay kit, Eagle Biosciences, Boston, MA) and estimated according to Imai et al. (2001).

#### Lipid Peroxides and Antioxidant Enzymes Levels

MDA level measured by colorimetric method using thiobarbituric acid reaction according to the method described by Draper et al. (1993). GSH was measured by the colorimetric method as described by Beutler et al. (1963). Determination of SOD activity was done according to Borgstahl and Oberley-Deegan (2018) using a spectrophotometer.

### Histopathological Analysis

Fixation of the adrenal gland specimens in 10% formal saline was performed. The specimens were processed to obtain paraffin blocks which were cut into sections of 5 μm thickness. The sections were stained with hematoxylin & eosin stain to illustrate the general adrenal gland architecture (Bancroft and Gamble, 2008). Paraffin adrenal gland sections were stained with Mallory trichrome stain to reveal the collagen fibers (Bancroft and Gamble, 2008). Paraffin sections were mounted on glass slides covered with pol-L-lysin. Sections were deparaffinized, dehydrated, and soaked in 10 M sodium citrate buffer (pH 6.0). For heating, sections were kept at 60°C for 10 min in a microwave oven. They were incubated with 0.1% sodium azide and 3% hydrogen peroxide to inactivate endogenous peroxidase and with 0.5% casein for 20 min at room temperature to block nonspecific staining. Sections were set in an antigen retrieval solution (0.01 M citrate buffer, pH 6.0) in a microwave oven at a temperature of 100°C at 600 W for 15 min. For caspase-3 immunostaining, a peroxidase-conjugated rabbit monoclonal antibody IgG (Cell Signaling Technology, Ipswich, USA) at dilution 1:200 was applied while for proliferating cell nuclear antigen (PCNA) immunostaining, mouse MAb anti-PCNA (clone PC10; DAKO) was kept in the sections at dilutions of 1:5,000. Moreover, for interleukin-6 immunostaining, interleukin-6 (IL-6) mouse monoclonal antibody (Sc-130,326; Santa Cruz Biotechnology Company, Dallas, TX, USA; ABC staining system: Sc-2017) was applied. Sections were incubated with primary antibodies overnight at room temperature then incubated with goat anti-mouse biotinylated IgG (no. B0529; Sigma-Aldrich, St. Louis, MO, USA) for caspase-3 and IL-6 immunostaining while incubated with polyclonal donkey anti-mouse (dilution 1:250 in PBS; Dako, Glostrup, Denmark) and extravidin-conjugated horseradish peroxidase (extravidin Peroxidase Staining Kit, Extra-2; Sigma-Aldrich, St. Louis, MO, USA) using a dilution of 1:200. Staining was obtained with diaminobenzidine (DAB; Sigma-Aldrich, St. Louis, MO, USA) substrate and washed using tap water, then was counterstained with hematoxylin. Slides were rinsed in distilled water till the sections get blue color.

Moreover, slides were dehydrated in ascending ethanol concentrations (70, 95, and 100%) for 5 min each concentration then were cleared using xylene and finally cover-slipped using histomount mounting solution (Lee et al., 2006; Kabiraj et al., 2015; Suker et al., 2017).

### Morphometric Study

For quantitative evaluation, 10 non-overlapping fields from each section were measured using a Leica DML B2/11888111 microscope supplied with a Leica DFC450 camera. The measured parameters were calculated using Image J software version K1.45. The mean thickness of each adrenal gland cortical zones was measured (zona granulosa, zona fasciculata, and zona reticularis). The measured data were undertaken using H&E sections (×200). The mean area percentage of collagen was measured in Mallory trichrome sections (×200). For quantitative immunohistochemical evaluation, the following parameters were calculated; percent of caspase-3 immunopositive intensity, number of PCNA immunopositive cells, and percent of IL6 immunopositive intensity. For quantitative immunohistochemical assessment, the adrenal tissue of all experimental groups was evaluated according to (a) intensity of the stain: mild, moderate, or strong; (b) staining pattern: cytoplasmic or nuclear; and (c) expression percentage: positive cells were counted, and more than 200 cells of the entire section were counted at 200× magnification.

### Transmission Electron Microscopy

Adrenal gland specimens were sliced into small pieces (1 mm^3^) and retained in 2.5% glutaraldehyde for 24 h for fixation. Semithin sections were obtained using an ultra-microtome (0.5–1-μm slices) and stained with toluidine blue staining for light microscopic examination. The adrenal blocks were trimmed, and then specific areas were chosen for ultrathin section preparations. A copper grid capsule was applied to each block. Lead citrate and uranyl acetate stains were applied for ultrathin section staining, then examined using a transmission electron microscope (SGS-Russia) SGS Egypt Limited LLC. in the Alexandria Medical Research Institute (Bancroft and Gamble, 2008).

### Statistical Analysis

Analysis was performed using SPSS version 22.0 (SPSS Inc., Chicago, IL, USA). Data were expressed as mean ± SD. ANOVA (F test) was used to compare several groups for normally distributed quantitative data. Test of homogeneity of variances was performed, and Tukey test *post hoc* analysis was used for assumed equal variance. Data were evaluated as statistically highly significant and significant at *p* < 0.001 and 0.05, respectively.

## Results

### Behavioral Tests

#### Forced Swim Test

**Figure 3:**
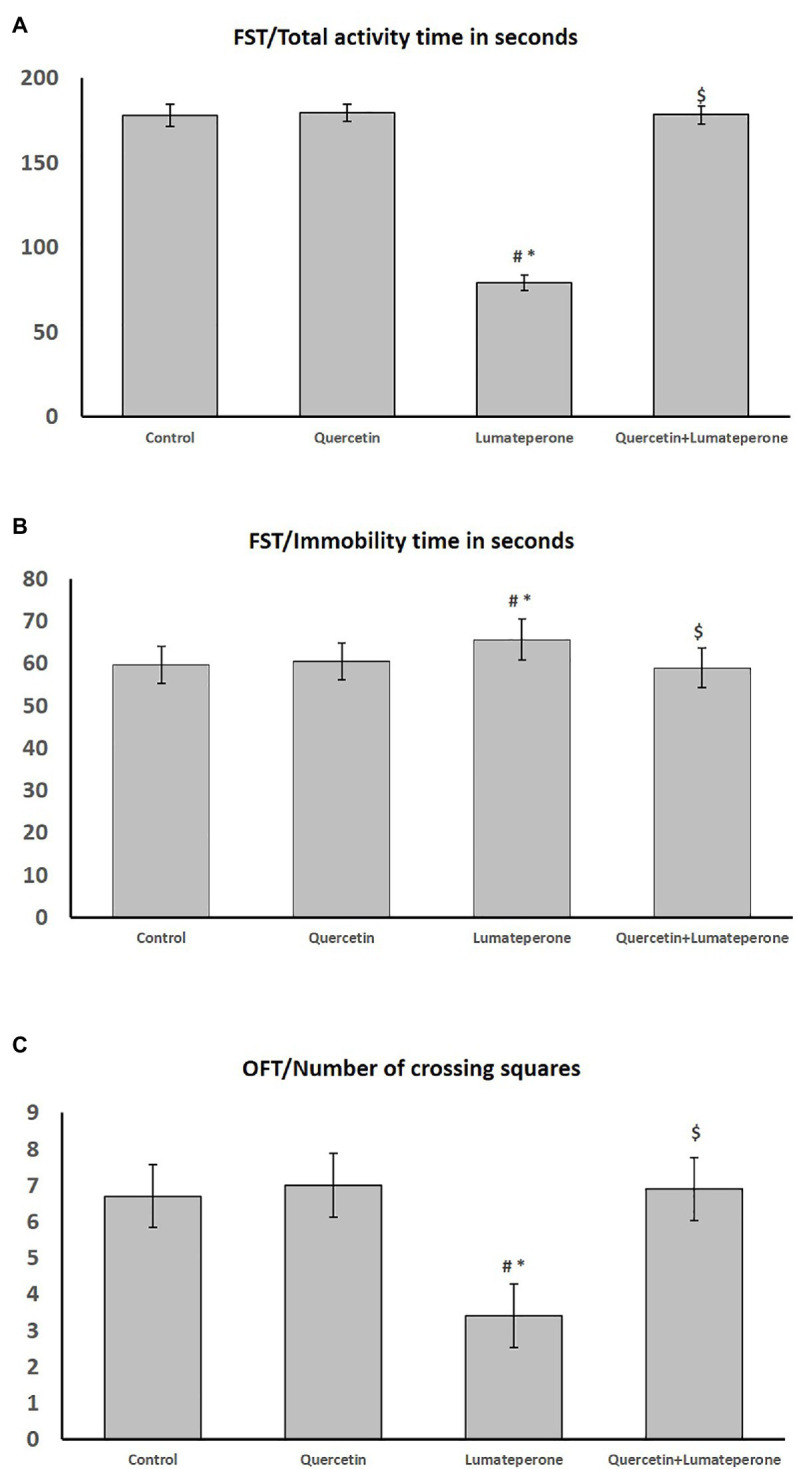
Behavioral test results among control, quercetin, lumateperone, and quercetin/lumateperone-cotreated group; **(A)** forced swim test (FST) total activity time in second among groups. **(B)** FST, immobility time in seconds among groups. **(C)** Open field test (OFT) number of crossing squares among groups. Data were expressed as mean ± SD (*n* = 10). One-way ANOVA: # < 0.05 vs. control; ∗ < 0.05631 vs. quercetin; $ < 0.05 vs. lumateperone.

As shown in Figures 3A,B, lumateperone-treated animals showed increased immobility time significantly (*p* < 0.001) while total activity time was significantly reduced (*p* < 0.001) compared with control group. Whereas quercetin/lumateperone-cotreated group immobility time was significantly decreased, total activity time was significantly increased compared to lumateperone-treated group, and it was insignificant compared to control.

#### Open Field Test

As shown in Figure 3C, lumateperone administration significantly reduced the number of crossing squares compared with control (*p* < 0.001). However, lumateperone combined with quercetin caused a significant increase in crossing squares compared with the corresponding value in the lumateperone group.

### Biochemical Analysis

Serum cortisol (ug/dL), DHEA (ng/ml), and Aldosterone level (ng/ml) in the lumateperone group were significantly lower (*p* < 0.001) than in the control group. In the quercetin-lumateperone-cotreated group, these hormones levels were significantly higher (*p* < 0.001) compared to the lumateperone group alone and insignificantly changed compared with control. While ACTH (pg/dL) was insignificantly changed among all groups (Table 1). GSH (nmol/ml) and SOD (U/ml) levels in the lumateperone-treated group was significantly lower (*p* < 0.001) compared to the control. In the quercetin/lumateperone cotreated group, these hormones were significantly higher (*p* < 0.001) compared to the lumateperone treated group and insignificant (*p* > 0.05) compared with the corresponding values in the control group (Table 1). MDA (nmol/ml) level in the lumateperone-treated group was significantly higher (*p* < 0.001) compared to the control group. In the quercetin/lumateperone-cotreated group, it was significantly lower (*p* < 0.001) compared with lumateperone-treated group and still significantly higher (*p* < 0.05) compared to its corresponding values in the control group (Table 1).

**Table 1:** Serum ACTH, cortisol, DHEA, aldosterone, MDA, GSH, and SOD in the control, quercetin-treated, lumateperone-treated and quercetin-lumateperone-cotreated groups.

	Control	Control + quercetin	Lumateperone	Quercetin + Lumateperone
Serum ACTH (pg/ml)	30.4 ± 0.65	30.71 ± 0.81	29.89 ± 0.58	29.82 ± 0.99
Serum Cortisol (ug/dL)	31.1 ± 0.975	30.71 ± 0.813	26.94 ± 0.895^#^ ^∗^	30.67 ± 0.31^$^
DHEA (ng/ml)	3.09 ± 0.21	3.03 ± 0.2	2.11 ± 0.22^#^^∗^	2.5 ± 0.44^$^
Aldosterone (ng/dL)	22.94 ± 1.23	22.5 ± 1.28	18.4 ± 1.43^#^^∗^	22.1 ± 1.58^$^
MDA(nmol/ml)	2.16 ± 0.30	2.3 ± 0.28	13 ± 1.84^#^^∗^	3.4 ± 0.8^#^ ^*^ ^$^
GSH (nmol/ml)	2.67 ± 0.43	2.95 ± 0.34	0.36 ± 0.12^#^^∗^	2.42 ± 0.35^$^
SOD (U/ml)	170.9 ± 3.7	171.8 ± 5.02	141.3 ± 3.26^#^^∗^	172.5 ± 5.46^$^

Data are expressed as mean ± SD (*n* = 10), one-way ANOVA.

# *p* < 0.05 vs. the control group.

∗ *p* < 0.05 vs. the control quercetin group.

$ *p* < 0.05 vs. the lumateperone group.

### Adrenal Gland Histological Assessment

#### Hematoxylin and Eosin

**Figure 4:**
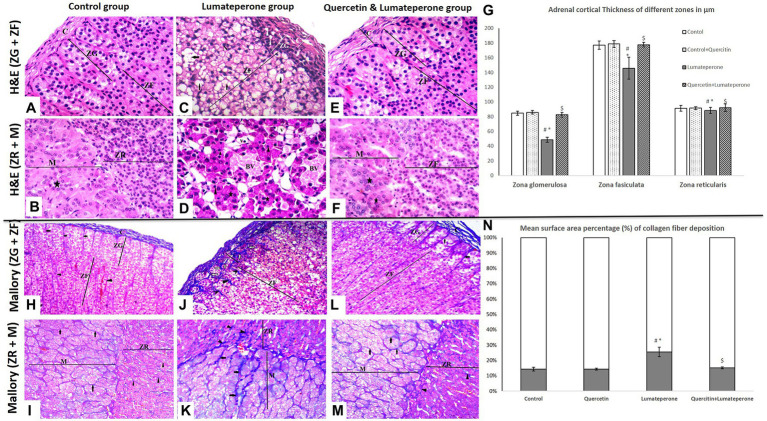
Adrenal Histopathology using hematoxylin and eosin (H&E×200) and Mallory trichrome (×200) staining. **(A)** A section of group I (control group) showing part of the adrenal cortex surrounded by a tissue capsule. Under the capsule, zona glomerulosa (ZG) cells are clustered. The next wider zone is zona fasciculata (ZF), with regular cell arrangement and foamy cytoplasm (H&E×200). **(B)** A section of group I (control group) showing part of the adrenal cortex, zona reticularis (ZR), contains eosinophilic cells arranged in cords near the adrenal medulla with chromaffin cells arranged in clusters (star; H&E×200). **(C)** A section of group III (lumateperone) adrenal cortex segments showing disorganized zona glomerulosa (ZG) cells with numerous intercellular spaces (arrowhead) and multiple deeply stained nuclei. Zona fasciculata cells (ZF) appeared separately disorganized, exhibit swollen, vacuolated cytoplasm (arrow), and several deeply stained nuclei. Note the apparent thick capsule (H&E×200). **(D)** A section of a group III (lumateperone) segment of the adrenal medulla with chromaffin cells arranged in clusters (star) with darkly stained small pyknotic nuclei (arrowhead). Note widening between medulla cells and congested blood sinusoids (BV; H&E×200). **(E)** Group IV (quercetin + lumateperone) section showing part of the adrenal cortex surrounded by a capsule of the connective tissue. Regular glomerulosa (ZG) and fasciculate zone (ZF) (H&E×200). **(F)** Group IV (quercetin + lumateperone) showing zone reticularis (ZR) has a typical normal appearance near the adrenal medulla with chromaffin cells in clusters (arrowhead; H&E×200). **(G)** Adrenal cortical thickness of different layer among groups. **(H)** Adrenal cortex of group I (control) showing usual regular capsule collagen fibers with regular fibers in extending trabeculae between ZG cells (short arrows) and zona fasciculata (arrowhead; Mallory’s trichrome×200). **(I)** A section of the adrenal gland group I (control group) showing part of the adrenal cortex, zone reticularis (ZR) with thin collagen fibers separating cells (arrowheads) near the adrenal medulla with thin collagen fibers separating chromaffin cell clusters (arrow; Mallory’s trichrome ×200). **(J)** Adrenal cortex segment of group III (lumateperone) displaying thick capsule collagen fibers and thick irregular trabeculae between ZG (short arrows) and cords of ZF (arrowhead; Mallory’s trichrome ×200). **(K)** A section of the adrenal gland group III (lumateperone) showing thick irregular trabeculae (arrowhead) between the zona reticularis (ZR) near the adrenal medulla with thick fibers separating the clusters of chromaffin cells (arrow; Mallory’s trichrome ×200). **(L)** A segment of group IV (quercetin + lumateperone) adrenal cortex showing nearly normal thick capsule (long arrow) and expanding trabeculae between ZG cells (short arrow; Mallory’s trichrome ×200). **(M)** A section of the adrenal gland group IV (quercetin + lumateperone) showing part of the adrenal cortex, zona reticularis (ZR) with thin collagen fibers between cords (arrowheads) near the adrenal medulla with thin collagen fibers separating chromaffin cell clusters (arrow; Mallory’s trichrome ×200). **(N)** Mean surface area percentage (%) of collagen fiber deposition among groups by morphometric analyses. Data were expressed as mean ± SD (*n* = 10). One-way ANOVA: # < 0.05 vs. control; ∗ < 0.05 vs. quercetin; $ < 0.05 vs. lumateperone. N.B. Control with quercetin group showed the same findings as the control group.

Adrenal sections of control group revealed a CT capsule with short, inadequate trabeculae. Cortex involved three zones: Zona Glomerulosa (ZG), which is a confined zone outlined of curves or adjusted masses of columnar cells (glomeruli) and disconnected by fenestrated blood vessels; Zona Fasciculata (ZF), which is a broad zone that is molded with straight lines of polyhedral cells with immense pale nuclei and vacuolated cytoplasm disengaged by fenestrated sinusoids (Figure 4A); and Zona Reticularis (ZR) which is a dainty zone made of spreading and anastomosing strings of polyhedral cells that contain barely fat beads. The medulla contains huge polyhedral Chromaffin cells with focal round nuclei and basophilic cytoplasm organized in gatherings. Sympathetic multipolar nerve cells were dispersed in chromaffin cells and lymphocytes around blood sinusoids and vessels (Figure 4B). Adrenal sections of the quercetin-treated group showed normal suprarenal histological structure, same as the control. Adrenal sections of the lumateperone-treated group displayed thickening and corrugation of the capsule encompassing the adrenal organ with separation of its strands. Zona glomerulosa was disordered with a significantly decreased thickness (Figure 4G) and pyknotic nuclei. The cells of zona fasciculata were messed up losing cord appearance with pyknotic nuclei and vacuolated cytoplasm (Figure 4C). Regardless of how the cell relationship of zona reticularis appeared with minimal histological changes, Chromaffin cells of the adrenal medulla showed vascular congestion, pyknotic nuclei, and necrotic chromaffin cells (Figure 4D). The adrenal glands of the animals treated with quercetin and lumateperone revealed histopathological stamped improvement compared to that is induced by lumateperone alone. The capsule was slightly thickened with cortical zones (ZG-ZF-ZR), revealing typical histological structure and thickness (Figure 4G). ZF scarcely exhibited vacuolations (Figures 4E,F). The adrenal medulla revealed an ordinary structure (Figure 4F).

#### Mallory Trichrome Stain

Mallory’s trichrome stained sections of the control group had the normal whole of collagen strands forming the capsule enveloping the organ with a negligible sum of collagen strands displaying up between the curves of zona glomerulosa (Figures 4H,I). Whereas lumateperone-treated group revealed a significant increase in mean percentage of collagen (Figure 4N) compared with the control group, including clear thickening of the capsule outlined the gland with wide zones between its collagens strands and thickening of collagen between all zones of the gland (Figures 4J,K). Lumateperone and quercetin-treated group had an average sum of collagen fibers shaping the capsule encompassing the gland with a negligible whole in between adrenal zone (Figures 4L,M).

#### IL-6 Immuno-Stain

**Figure 5:**
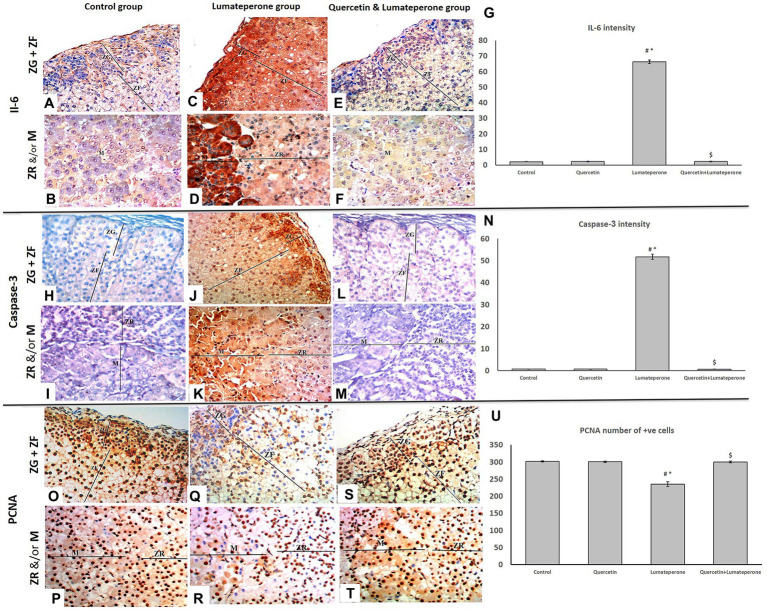
Adrenal immune histochemistry. **(A)** A section of group I (control) showing mild cytoplasmic reaction for IL-6 of adrenal cortical cells of both ZG and ZF (IL-6×200). **(B)** A section of group I (control) showing mild cytoplasmic reaction for IL-6 of adrenal cells of both ZR and medulla (IL- 6×200). **(C)** Group III (lumateperone) showing extensive cytoplasmic reaction for IL-6 of adrenal cortical cells of ZG and the strong cytoplasmic reaction of ZF (IL-6×200). **(D)** Group III (lumateperone) showing mild cytoplasmic reaction for IL-6 of adrenal cortical cells of ZR and the strong cytoplasmic reaction of the medulla (IL-6×200). **(E)** Group IV (quercetin + lumateperone) showing mild cytoplasmic reactions for IL-6 of adrenal cortical cells of both ZG and ZF (IL-6×200). **(F)** Group IV (quercetin + lumateperone) showing mild cytoplasmic reaction for IL-6 of adrenal medulla (IL-6×200). **(G)** IL-6 intensity among groups analyzed by morphometry **(H)** Group I (control) showing negative cytoplasmic reactions for caspase-3 of adrenal cortical cells of both ZG and ZF (Caspase-3×200). **(I)** A section of group I (control) showing negative cytoplasmic reaction for caspase-3 of adrenal cortical cells of both ZR and medulla (caspase-3×200). **(J)** Group III (lumateperone) showing extensive cytoplasmic reaction for caspase-3 of adrenal cortical cells of ZG and moderate cytoplasmic reactions of ZF (caspase-3×200). **(K)** Group III (lumateperone) showing mild cytoplasmic reactions for caspase-3 of adrenal cortical cells of ZR and the strong cytoplasmic reaction of the medulla (caspase-3×200). **(L)** Group IV (quercetin + lumateperone) showing negative cytoplasmic reaction for caspase-3 of adrenal cortical cells of both ZG and ZF (caspase-3×200). **(M)** A section of group IV (quercetin + lumateperone) showing negative cytoplasmic reaction for caspase-3 of adrenal cortical cells of both ZR and medulla (caspase-3×200). **(N)** Caspase-3 intensity among groups analyzed by morphometry. **(O)** A PCNA stained section of group I, showing the positive nuclear reaction of most cells of adrenal cortical cells of both ZG (arrow) and ZF (arrowhead; PCNA ×200). **(P)** Group I, showing PCNA-positive nuclear reaction of adrenal cells of both ZR (arrowhead) and medulla (arrow; M; PCNA ×200). **(Q)** A section of PCNA stained section of group III (lumateperone) showing the negative nuclear reactions of many cells of adrenal cortical cells of both ZG (arrow) and ZF (arrowhead; PCNA ×200). **(R)** Group III (lumateperone) showing PCNA-negative nuclear reaction of adrenal cells of both ZR (arrowhead) and medulla (arrowhead; PCNA ×200). **(S)** A PCNA stained section of group IV (quercetin + lumateperone) showing a positive nuclear reaction of most cells of adrenal cortical cells of both ZG (arrow) and ZF (arrowhead; PCNA ×200). **(T)** Group IV (quercetin + lumateperone) showing PCNA-positive nuclear reaction of adrenal cells of both ZR (arrowhead) and medulla (arrow; PCNA ×200). **(U)** PCNA number of +ve cells among groups analyzed by morphometry. Data of morphometry were expressed as mean ± SD (*n* = 10). One-way ANOVA: # < 0.05 vs. control; ∗ < 0.05 vs. quercetin; $ < 0.05 vs. lumateperone. N.B. Control with quercetin group showed the same findings as the control group.

Adrenal sections stained with IL-6 immuno-stain of the control group showed mild response to-IL-6 immunoreactivity (Figures 5A,B). Sections of the lumateperone-treated group uncovered a significant solid positive cytoplasmic reaction to IL-6 immunoreaction (Figure 5G) in most of the cells (Figures 5C,D), although the group of lumateperone and quercetin revealed mild anti-IL-6 immunoreaction (Figures 5E,F).

#### Anti-caspase-3

Adrenal sections stained with Caspase 3 immuno-stain of the control group are negatively hostile to Caspase immune reactivity (Figures 5H,I). Sections of the lumateperone-treated group uncovered solid positive cytoplasmic response (Figure 5N) to Caspase immunoreaction in most of the cells (Figures 5J,K), whereas the group of lumateperone and quercetin appeared negative anti-Caspase immunoreaction (Figures 5L,M).

#### Proliferating Cell Nuclear Antigen

Adrenal sections stained with anti-PCNA counter antibodies appeared as solid positive immune-reactive cores within the control (Figures 5O,P), whereas the group treated with lumateperone uncovered moderate response (Figure 5U) plus many cells with negative immune reaction (Figures 5Q,R). In comparison, lumateperone and quercetin uncovered a positive response (Figures 5S,T).

#### Electron Microscopic Results

**Figure 6:**
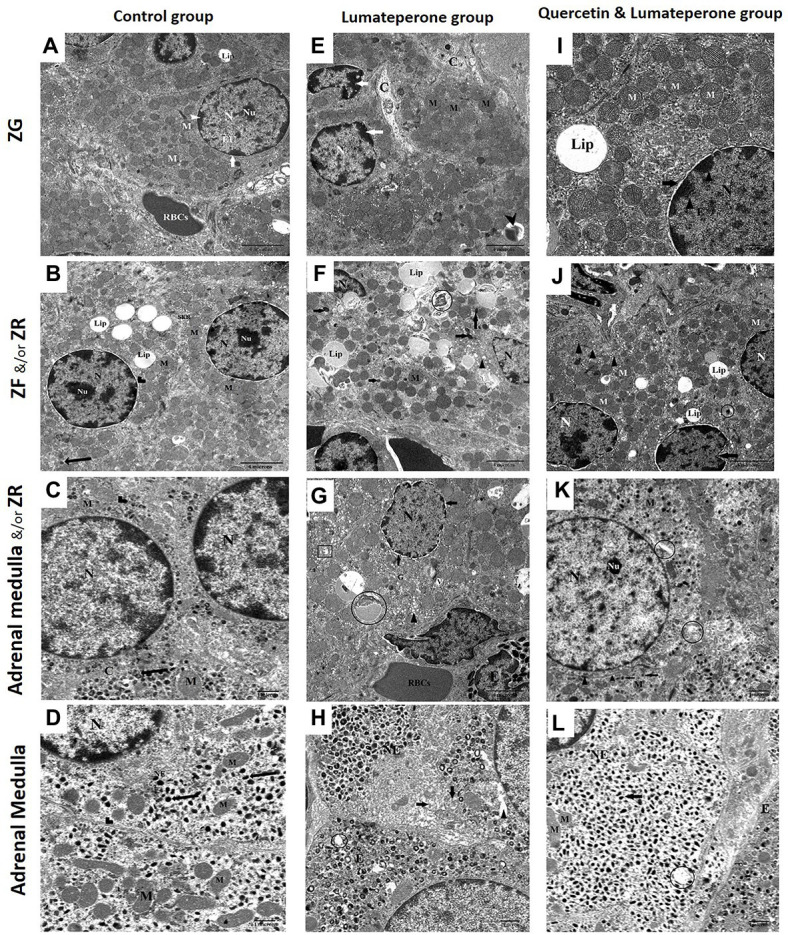
Electron microscopy **(A)** Transmission electron micrograph of the adrenal gland in control group showing a more or less normal cell in ZG of suprarenal cortex with abundant lipid droplets (Lip), rounded nuclei surrounded by the nuclear envelope (arrow), having prominent nucleolus (Nu), heterochromatin (arrowhead) and euchromatin. Note: blood capillary contains RBCs, capillary typical ultrastructure with a normal endothelial wall (×8,000). **(B)** Control group showing most probably normal two adjacent cells in ZF and ZR of the suprarenal cortex. Cell in ZF demonstrating multiple lipid droplets (Lip), rounded nuclei surrounded by the nuclear envelope (arrowhead), nucleolus (Nu), and a lysosome (arrow). While ZR cells illustrating normal smooth endoplasmic reticulum (SER), mitochondria, round nucleus possessing peripheral heterochromatin and nucleolus (Nu; ×8,000). **(C)** Adrenal gland medulla of control group showing normal epinephrine with medium electron-dense granules (arrow), regular rounded nucleus, mitochondria, and intact cell junction (arrowhead), homogenous cytoplasm (×17,500). **(D)** Control group showing normal norepinephrine (NE) with very electron-dense granules (arrow) of the medulla, regular rounded nucleus, mitochondria, and intact cell junction (arrowhead; ×17,500). **(E)** Lumateperone-treated group showing ZG with severe infiltration with collagen fiber, lysosome (arrowhead) irregular nucleus with peripheral condensation of chromatin (arrow) and mitochondria (M; ×8,000). **(F)** Lumateperone-treated group showing ZF with massive accumulation and fusion of lipid droplet (Lip), distortion of lipofuscin drops (circle), pycnotic nucleus, increase in the number of lysosomes (arrow), dilated SER (arrowhead), and mitochondria (M; ×8,000). **(G)** Lumateperone-treated group showing eosinophilic infiltration of ZR, cells of ZR showing dilated SER (arrowhead), dilated perinuclear membrane (arrow), cytoplasmic vacuolations, irregular nucleus, degenerated mitochondria (rectangle), lipofuscin drops (circle) and congested blood capillary with RBCs. Note the normal appearance of Golgi (×8,000). **(H)** Adrenal gland medulla of lumateperone-treated group showing norepinephrine cell (NE) with a decrease in the number of secretory granules, partial lysis of cristae of mitochondria (arrow), rarified cytoplasm (arrowhead), also depletion of some secretory granules of epinephrine cell with degeneration of their mitochondria (curved arrow; (×17,500). **(I)** Quercetin and lumateperone-treated group showing more or less normal cells in ZG of suprarenal cortex with one large lipid droplet (Lip), rounded nuclei surrounded by the slightly dilated nuclear envelope (arrow), having heterochromatin (arrowhead) and euchromatin. Multiple normal mitochondria (M; ×8,000). **(J)** Quercetin and lumateperone-treated group showing the number of cells in ZF of suprarenal cortex with slightly irregular in the nucleus, mild indented nuclei of one of them (arrow), normal SER (arrowhead), different degree of saturation of lipid droplet (Lip), normal mitochondria and lysosome (circle; ×8,000). **(K)** Adrenal medulla of quercetin and lumateperone-treated group showing epinephrine with medium electron-dense granules (arrow), focal area of rarified cytoplasm (circle), regular rounded nucleus, nucleolus (Nu), normal mitochondria, and intact cell junction (arrowhead; ×17,500). **(L)** Transmission electron micrograph of the adrenal gland (medulla) in quercetin + lumateperone-treated group showing improvement in norepinephrine cell (NE) with electron-dense secretory granule (arrow), rounded regular nucleus, mitochondria, while epinephrine cell with medium electron-dense secretory granule (arrowhead). Note: few mitochondria in norepinephrine cells are degenerated (circle; ×17,500). N.B. Control with quercetin group showed the same findings as the control group.

Transmission electron microscopic finding of the adrenal gland of control group appeared ordinary cells in ZG of suprarenal cortex with plenteous lipid beads, rounded nucleus encompassed by a nuclear envelope with conspicuous nucleolus with peripheral heterochromatin. Blood capillary contains RBCs were typical ultrastructure with a normal endothelial wall (Figure 6A). The cell in ZF illustrated different lipid beads, round nucleus encompassed by a nuclear envelope, nucleolus, and lysosomes. In comparison, ZR cells outlined ordinary smooth endoplasmic reticulum, mitochondria, rounded nucleus having fringe heterochromatin, and nucleolus (Figure 6B). Adrenal medulla epinephrine cells revealed medium electron-dense granules, customary round nucleus, mitochondria, and intact cell junction. Norepinephrine cells illustrated exceptionally electron-dense granules, normal round nucleus, mitochondria, and intact cell junction (Figures 6C,D).

Adrenal gland lumateperone-treated group revealed ZG cells with extreme infiltration with collagen fibers and irregular nucleus with fringe condensation of chromatin (Figure 6E). Cells of ZF showed an enormous collection and combination of lipid bead, twisting of lipofuscin beads, pyknotic nucleus, increment in the number of lysosomes, and expanded SER (Figure 6F). ZR showed eosinophilic invasion, expanded SER, widened perinuclear envelope, cytoplasmic vacuolation, irregular nucleus, degenerated mitochondria, lipofuscin beads, and congested blood capillary with RBCs with the typical appearance of Golgi (Figure 6G). Adrenal medulla uncovered norepinephrine cells with diminishing in the number of secretory granules, halfway lysis of cristae of mitochondria, rarified cytoplasm, moreover exhaustion of a few secretory granules of epinephrine cell with degeneration of their mitochondria (Figure 6H).

The adrenal gland treated with lumateperone and quercetin uncovered cells of ZG with a round nucleus and marginally widened nuclear envelope and typical mitochondria (Figure 6I). Cells in ZF appeared with somewhat irregular nucleus, a mellow indented nucleus of one of them, typical SER, distinctive degree of immersion of lipid bead, typical mitochondria, and lysosome (Figure 6J). Adrenal medulla epinephrine cells revealed medium electron-dense granules, central region of rarified cytoplasm, normal round nucleus, nucleolus, ordinary mitochondria, and intact cell junction (Figure 6K).

Norepinephrine cells showed up with electron thick secretory granule, standard nucleus, and mitochondria. Some mitochondria in norepinephrine cells were degenerated (Figure 6L).

## Discussion

The present study used Guinea pigs since their human counterparts exhibit higher homology and pharmacological responses than murids (Wicke et al., 2007). The current study aimed to identify the effects of administering an atypical antipsychotic drug, lumateperone alone or with quercetin, on animal behavior and adrenal gland functions. In the current study, lumateperone-treated group showed a significant decrease in the total activity time, increased immobility time in the forced swim test, and decreased crossing in the open field test. This is consistent with the FDA Caplyta approval report of adverse effects of lumateperone, including thin appearance, ataxic and hypoactive behavior, splayed gait, splayed posture, and limited use of the hind legs [Caplyta (lumateperone), 2018], somnolence/sedation, and fatigue (Correl et al., 2020). On the other hand, quercetin/lumateperone treatment increased total activity time and decreased immobilization time in forced swim tests, and increased crossing in the open field test. The behavioral despair test (or Porsolt forced swimming test) results have been interpreted as measuring animal susceptibility to depressive-like behavior and negative mood depending on the animal response to the threat of drowning and to passive behavior that withdraws the animal from coping with stressful stimuli actively (Yankelevitch-Yahav et al., 2015). The open field result is a measure of the locomotor as well as the exploratory behavior. This means that quercetin coadministration with lumateperone decreased the susceptibility to negative mood and increased the exploratory behavior compared to lumateperone alone.

The adrenal gland structure and its hormonal secretion in this study showed a significant reduction with lumateperone treatment compared to control. The thickness of the adrenal cortical layer as ZG, ZF, and ZR was significantly reduced. This was associated with a reduction in serum levels of cortisol, aldosterone, and DHEA hormones. Despite that, the ACTH was not significantly changed. This was in concordance with Ryan et al. (2004), who suggested different mechanisms for elevated cortisol in SCZ patients other than ACTH mediated mechanism. Second-generation antipsychotics showed significantly decreased cortisol levels in healthy individuals than first-generation (Cohrs et al., 2006). This effect may be due to the blocking of serotonergic receptors by the atypical drugs. The observed downregulation of the pituitary–adrenal axis may be clinically relevant for the impact on depressive symptoms and cognitive functioning (Cohrs et al., 2006).

Cortisol has been demonstrated to be a mortality indicator in patients with depression (Chan et al., 2010). An exhausted cortisol production might lead to a hyper-immune state as acute immune responses continue unopposed (McEwen, 1998). Increased IL-6 expression in the adrenal gland with lumateperone treatment was associated with suppressed animal activity, and its decreased expression with quercetin cotreatment was associated with increased animal activity. This was in concordance with Ting et al. (2020), who stated that IL-6 activity increased in depression, and therefore, its suppression is required to proceed with clinical recovery. IL-6 is a mediator in the immune system’s interaction with the HPA axis, and it has been well developed in the rat adrenal gland (Judd and MacLeod, 1992). In all zones of the adrenal cortex, IL-6 works on steroidogenesis (Päth et al., 1997).

IL-6 also interacts directly with the adrenal medulla chromaffin cells and can modulate the synthesis of both catecholamine and neuropeptide chromaffin cells, thereby influencing the stress response of the adrenal cells (Jenkins et al., 2016). However, IL-6 has two different signaling pathways: either *via* the membrane-bound receptor causing protective effects or through “trans-signaling” provoking pathologic effects (Kalkman, 2019). The trans-signaling pathway could explain the increased IL-6 expression with the reduction in cortisol level and the associated adrenal gland pathology in the lumateperone-treated group.

ACTH is a key component of the HPA axis regulating homeostasis during stress. ACTH binds to melanocortin (MC) 2 receptors (MC2R), producing cortisol and, to some extent, aldosterone. Low expression of these receptors coincides with adrenal cell apoptosis, whereas enhanced expression can contribute to cell vitality. The ACTH effect on zona fasciculate cells mediating cortisol synthesis depends on complex intra-adrenal interaction networks such as chromaffin cells in the adrenal medulla, numerous immune system cells including monocytes/macrophages, mast cells, lymphocytes, vascular endothelial cells, and adipocytes inside and around the gland (Angelousi et al., 2000). So adrenal gland detected pathological changes associated with lumateperone treatment, explains its resistance to ACTH stimulatory effect.

The decreased DHEA level with lumateperone in our study is associated with histopathological affection of zona reticularis that is in line with the preclinical results for evaluating lumateperone. It is noteworthy that oral administration of lumateperone to male and female rats adversely affects male and female fertility and causes estrus cycle irregularities. Further, lumateperone causes testicular toxicity in rats at a large dose and decreases in mating and fertility indices at all doses when treated males were paired with treated females [Caplyta (lumateperone), 2018]. Moreover, aldosterone hormone showed a significant decrease with lumateperone treatment associated with a significant reduction in ZG with marked IL-6 infiltration. This may explain the lumateperone treatment-related effects [e.g., hypoactivity, lethargy, cardiac conduction problems, decreased body weight, and dehydration; Caplyta (lumateperone), 2018], as aldosterone hormone is responsible for electrolyte balance and blood volume, and pressure regulation.

In current study, the electron microscopy results of the adrenal medulla of control and quercetin/lumateperone-treated group showed medium electron-dense granules in epinephrine cells and thick secretory granules in norepinephrine cells denoting normal secretory activity, compared to the lumateperone-treated group. The latter revealed diminish in the number of secretory granules with the exhaustion of a few secretory granules of epinephrine cells and degeneration of their mitochondria. Epinephrine is an important factor for cortisol secretion *via* an alpha-adrenergic mechanism (Mokuda et al., 1992), a proposed lumateperone blocking action site. The deterioration in the adrenal gland histological structure with increased inflammatory marker, cell apoptosis, and collagen deposition in the lumateperone-treated group was in concordance with the concerns of internal FDA pathologists, which have been made on the drug. In particular, concerning the accumulation of pigmented material observed on gross necropsy and often located within parenchymal cells and infiltrating macrophages and other mononuclear inflammatory cells in multiple organs, including the adrenals, and occasionally in the extracellular space [Caplyta (lumateperone), 2018]. It was suggested that these pigmented depositions are the aniline metabolites of lumateperone, and they are trapped in lysosomes, with subsequent lysosomal dysfunction. The electron microscopic results of the adrenal cortex in the lumateperone group supported their opinion as there was an increment in the number of lysosomes in ZF, eosinophilic invasion in ZR, congested blood capillaries, accumulation of lipid droplets, and mitochondrial alteration associated with other manifestation of cellular inflammation and apoptosis that may lead to inflammation and degeneration of adrenal cells. The lumateperone group has adrenal lysosomal accumulation that can disrupt lysosomal homeostasis [Caplyta (lumateperone), 2018] and the eventual loss of cell viability, cellular toxicity, and inflammation (Lenz et al., 2018).

The findings from this study suggest that quercetin treatment decreased the expression of inflammatory marker IL-6, Caspase-3 apoptotic marker, and increased PCNA, denoting increased cell proliferation. These restored the thickness of the adrenal cortices and structure of the medulla, which explains the associated normalization of hormonal levels of cortisol, aldosterone, and DHEA.

Kaczmarczyk et al. (2004) assessed PCNA expression as a marker for cellular proliferation in Guinea pigs’ adrenal glands and concluded that the balance between proliferation and apoptosis is crucial for its normal function. Increased PCNA expression with quercetin indicates increased regenerative capacity of adrenal tissue suppressed with lumateperone due to histiocytic inflammation and degenerative damage. Quercetin coadministration lowered the MDA level due to its scavenging ability (Prochazkova et al., 2011). It is considered one of the most potent antioxidants *in vivo* and *in vitro*, reducing reactive oxygen species (Gao et al., 2014). It also stops the propagation of lipid peroxidation and increases glutathione (GSH) levels (Ansari et al., 2009). Restoration of the oxidant/antioxidant balance, reduction of MDA, and increased GSH and SOD antioxidant levels also play a crucial role in quercetin prophylactic effect. GSH plays a key role in detoxifying lipid peroxides and defense against reactive oxygen species (Ribas et al., 2014). GSH is not merely involved in antioxidant defense, but it is also involved in regulating cellular metabolic function, DNA and protein synthesis, signal transduction, cell proliferation, and apoptosis through modulating net oxidative stress and autophagy (Aquilano et al., 2014).

SOD is a key regulator of oxidative stress, catalyzing the superoxide radicles into oxygen molecules and hydrogen peroxide (Halliwell and Gutteridge, 2007). SOD levels were greatly boosted and restored by quercetin, suggesting that it may protect against oxidative damage.

Despite quercetin’s ability to induce DNA damage and cancer cell death by its prooxidant properties at high dose (Vargas and Burd, 2010), However in therapeutic doses, it prevents DNA damage and cell apoptosis, as evidenced in the current study by reduced cellular IL6, caspase-3, and increased PCNA together with its ability to restore the oxidative reduction balance. Li et al. (2019) found that quercetin, with a dose double of that in the current study, promoted reversal of neuronal damage and improved cognition by lowering MDA and increasing SOD, CAT, and GSH levels.

## Conclusion and Clinical Implications

Lumateperone market entry provides a revolutionary approach to treat SCZ with both new mechanisms of action and a markedly reduced side effects profile. Lumateperone is a novel second-generation antipsychotic proved to decrease adrenal gland functions with degenerative inflammatory effects on its ultrastructure and increased expression of IL-6, caspase-3, and reduced PCNA associated with hypoactive behavior. On the other hand, quercetin is a potent antioxidant, anti-inflammatory agent that reduced lipid peroxidation marker, MDA, and increased antioxidants as GSH and SOD. Also, it reduced adrenal cell apoptosis and induced DNA damage by lowering inflammatory markers (IL-6 and caspase-3), resulting in adrenal gland structural and functional improvement, and finally restored the normal level of animal activity. So, quercetin represents a new prospect for alleviating the possible side effects of lumateperone.

Thus, our results indicate the robustness of the protective effect of quercetin against the possible unwanted side effects of lumateperone. This may be an appropriate combination for patients with treatment-resistant forms of psychotic disorders. A combination of novel antipsychotic drugs and modulating drugs that might improve negative schizophrenic symptoms and cognitive function and thereby social functioning and quality of life. However, further research is needed to determine the safety of this combination using a well-established SCZ animal model.

## Study Limitations and Future Perspectives

Our study comes with some limitations. First, it was better to use an animal model of SCZ to assess the actual effect of the quercetin and lumateperone. However, the current study focused on the effect of lumateperone on the adrenal gland without studying its therapeutic potential as a multi-target-directed ligand and a multifunctional modulator of the serotoninergic system with a possible precognitive antipsychotic, antidepressant, and anxiolytic properties. Another limitation stemmed from using a generic available quercetin instead of its pure powder. The reason for this is the Movement Conditioned Order (MCO) applied nationwide for the pandemic C19 with difficulty in shipping along with the high expenses of its pure powder. However, the quercetin capsules do not contain too many impure chemical ingredients that may affect our study. A third limitation to the current study was registering animal behavior in forced swim tests and open field tests manually as we do not have access to computerized software which is expensive. We used manual observation and recording, which may be subjective with a bias. However, all efforts were done to avoid any bias by a blinded researcher who is unaware of the grouping or experimental designs.

## Data Availability Statement

The original contributions presented in the study are included in the article/supplementary material, further inquiries can be directed to the corresponding author.

## Ethics Statement

The animal study was reviewed and approved by Institutional Animal Care and Use Committee (IACUC), Menoufia University, Egypt (MUFS/F/HI/2/20).

## Author Contributions

All authors listed have made an equal substantial, direct and intellectual contribution to the work, and approved it for publication.

### Conflict of Interest

The authors declare that the research was conducted in the absence of any commercial or financial relationships that could be construed as a potential conflict of interest.

## Abbreviations

ACTH: Adrenocorticotropic hormone
DHEA: Dehydroepiandrosterone acetate
GSH: Glutathione
HPA: Hypothalamic–pituitary–adrenal axis
IL-6: Interleukin-6
MDA: Malondialdehyde
PCNA: Proliferating cell nuclear antigen
ROS: Reactive oxygen species
SOD: Superoxide dismutase
